# Histatin 5 Metallopeptides and Their Potential against *Candida albicans* Pathogenicity and Drug Resistance

**DOI:** 10.3390/biom11081209

**Published:** 2021-08-13

**Authors:** Gabriela Vieira Silva Zolin, Fauller Henrique da Fonseca, Carolina Reis Zambom, Saulo Santesso Garrido

**Affiliations:** UNESP—Department of Biochemistry and Organic Chemistry, Institute of Chemistry, Sao Paulo State University, Araraquara 14800-060, SP, Brazil; gabriela.zolin@unesp.br (G.V.S.Z.); fauller.henrique@unesp.br (F.H.d.F.); carolina.zambom@unesp.br (C.R.Z.)

**Keywords:** *Candida albicans*, Histatin 5, antifungal peptides, metallopeptides

## Abstract

Usually caused by *Candida* *albicans*, buccal candidiasis begins with the morphological transition between yeast and hyphal cells. Over time and without the correct treatment, it can be disseminated through the bloodstream becoming a systemic infection with high mortality rates. *C. albicans* already shows resistance against antifungals commonly used in treatments. Therefore, the search for new drugs capable of overcoming antifungal resistance is essential. Histatin 5 (Hst5) is an antimicrobial peptide of the Histatin family, that can be found naturally in human saliva. This peptide presents high antifungal activity against *C. albicans*. However, Hst5 action can be decreased for interaction with enzymes and metal ions present in the oral cavity. The current work aims to bring a brief review of relevant aspects of the pathogenesis and resistance mechanisms already reported for *C. albicans*. In addition, are also reported here the main immune responses of the human body and the most common antifungal drugs. Finally, the most important aspects regarding Histatin 5 and the benefits of its interaction with metals are highlighted. The intention of this review is to show the promising use of Hst5 metallopeptides in the development of effective drugs.

## 1. Introduction

The resistance to antimicrobials is currently one of the biggest health problems in the world. Several factors contribute to the rise of cases of infections caused by multi-drug resistant microorganisms: the increase in patients with suppressed immune systems due to diabetes, cancer and AIDS; the higher number of patients who need invasive treatments such as hemodialysis, venous catheters, transplants and mechanical ventilation; and the higher prevalence of treatment with steroids, hyperglycemia, use of broad-spectrum antibiotics and antifungals in subinhibitory concentrations [1,2,3,4,5].

After the development of antibiotics, with the discovery of penicillin in the first half of the 20th century, the interest in fungal infections increased. Infections caused by opportunistic yeast species, such as the *Candida* genus, are among the most recurrent ones and may present as topical infections in the oral cavity, genitourinary tract, or skin. The *Candida* genus also appears to cause systemic infections, which can spread through the blood reaching different organs [2,3,4,5,6,7].

In some cases, infections caused by the *Candida* genus could become long-lasting and easily progress to severe cases that can increase healthcare costs and extended hospital stays. Besides that, it is important to emphasize that prolonged hospital treatment results in the use of second- or third-line drugs which have higher chances of treatment failure [7]. The use of antimicrobial agents has been exponential since its development. In the period 2000–2010, BRICS countries (Brazil, Russia, India, China, and South Africa) were responsible for three-quarters of the global increase in antibiotic consumption [8,9]. The unrestrained use of antimicrobial agents contributes to the increase in resistance by microorganisms to commonly used drugs, affecting global health severely. 

It is estimated that around 700,000 deaths occur annually worldwide because of antimicrobial resistance, at a cost of around 9 billion euros only in Europe [7,8,10]. If no action is taken, predictions indicate that antimicrobial resistance could cause up to 10 million deaths per year, and cost trillions of dollars by 2050 [8,11]. This topic was discussed by the UN General Assembly in 2016, and antimicrobial resistance is considered today as one of the top 10 global health problems facing humanity [8,12,13].

Studies have already shown that pandemic caused by the SARS-CoV-2 virus increases the susceptibility of critically ill patients to fungal diseases such as pulmonary aspergillosis, pneumocystis pneumonia, mucormycosis and oral candidiasis [14,15,16,17,18,19]. The frequent use of mechanical respirators, catheters and complete parenteral nutrition in severe cases of COVID-19, may also be associated with an increase in mucormycosis incidence. Mucormycosis is a rare and aggressive opportunistic fungal infection, characterized by infarction and necrosis of host tissues, caused by *Rhizopus* gender. Factors such as low oxygen rate, high iron levels, high glucose levels and low phagocytic activity of the immune system caused by COVID-19 contribute to the infection’s development [20,21,22,23,24,25]. The number of *Candida* infections is also affected by prolonged clinical treatments, due to the formation of biofilms on equipment surfaces [15]. The biofilm formation can promote the spread of the fungus causing systemic infections. 

Data show that about 90% of all cases of invasive infections are caused by *Candida albicans*, *Candida tropicalis*, *Candida parapsilosis*, *Candida glabrataand Candida krusei* [1,3,26]. An increasing incidence of *C. parapsilosis*, *C. glabrata* and *C. tropicalis* species was observed in recent years, and all of the species have been showing some type of antifungal resistance. The super-resistant species, *Candida auris*, has already had its incidence reported in different countries. Some studies have shown the influence of COVID-19 in antimicrobial resistance, mainly in fungal infections. These studies highlight the high occupancy rate of hospitals and the high number of patients in intensive care units as a critical factor for the rise in antimicrobial resistance because they are exposed to multidrug-resistant organisms, such as *C. auris* [14,27,28,29]. *C. auris* is found mainly in hospitals, and is resistant to almost all available treatments, being mainly associated with systemic infections, with high mortality rates [30]. Despite that, the predominant species in topical and systemic infections are still *Candida albicans* [31,32,33]. 

*C. albicans* are eukaryotic microorganisms like human cells, and this factor reduces the number of selective targets that can be explored and impair the development of new antifungal agents. For this reason, the search for alternative therapies such as active molecules of natural products, polymeric materials, synthetic agents and bioactive peptides is important [2,34]. 

In this context, bioactive peptides present in the human body become interesting molecules since they have low cytotoxicity with less chance of side effects [2]. These peptides are considered key elements to the immune system, being often the first immune response against microbial infections. Some peptides isolated from the human body with antimicrobial activity are classified as antimicrobial peptides (AMPs). In general, they are characterized as small basic cationic peptides derived from proteins that exhibit antimicrobial activity. These ones include lactoferrins, defensins, cathelicidins and histatins [35]. 

A member of the Histatin family, Hst5 is a proteolytic fragment of Histatin 3, with 24 amino acids. It is produced and secreted by the sublingual, parotid and submandibular glands and is found naturally in human saliva. Hst5 is the peptide with the greatest antifungal activity in the family, and it can inhibit the growth of yeasts and hyphae of *C. albicans*. However, the antifungal activity of Hst5 is lower in vivo than in vitro because it may be influenced by external agents such as proteins, metals, salts and proteases found in saliva that reduce its antifungal activity [35]. The decrease in antifungal activity due to interferents presents in saliva environment, as well as the already existing resistance of the fungus to the peptide, requires new strategies for the improvement of Hst5 antifungal activity. Among such strategies are the synthesis of Hst5 fragments and the development of metal complexes formed with the peptide, which could increase the activity against *C. albicans* [36,37,38,39,40,41,42]. 

Thus, the aim of this work is to review concepts about the morphology, pathogenicity, and virulence factors of *C. albicans* as well as the human body’s immune response against these microorganisms. In addition, this review proposes to highlight existing antifungal treatments, their mode of action and the mechanisms of resistance already reported, to thus present the characteristics and mechanisms of antifungal action of Hst5. Finally, we report here the works that were carried out in order to increase the action of Hst5 through an association between peptides and metallic ions. This strategy could generate new molecules derived from Hst5, with greater antifungal potential, making them a possible treatment against fungal infections caused by *C. albicans* and other species.

## 2. *Candida albicans*: Morphology and Virulence

The *Candida* genus includes more than 200 species of single-celled eukaryotic organisms. These organisms have a cell wall composed of sterols outside the plasma membrane and have an ideal growth temperature around 37 °C. Furthermore, they are able to metabolize glucose in both aerobic and anaerobic conditions [1,26]. Several species such as *Candida albicans*, *Candida dubliniensis*, *Candida glabrata*, *Candida guilliermondii*, *Candida kefyr*, *Candida krusei*, *Candida parapsilosis*, *Candida tropicalis* and *Candida viswanathii* can be found naturally in the oral, gastrointestinal and genitourinary tracts of 40–60% of the population. *C. albicans* is the most recurrent and it appears in the commensal form of yeast, being harmless. The interaction between the fungal cells and the host immune systems is what maintains it is in the commensal form [43]. 

However, sometimes, the environmental conditions can allow the growth of filaments in the fungal cell, enabling the morphological transition to the hyphae form, which is the virulent morphology of *C. albicans* [2,3,33,44]. During infection, yeast cells are primarily responsible for the spread of the fungus, while hyphal cells are predominant in the mechanisms of invasion and acquisition of nutrients. The morphological transitions that allow the hyphae growth in *C. albicans* cells are induced after initial contact with the host cell and by the expression of different proteins. Some of these proteins are: Hwp1, Als3, secreted aspartic proteases Sap4, Sap5, Sap6, hypha-associated proteins Ece1, Hyr1 and contribute to adhesion and invasion mechanisms [3,26,33,45,46]. The genes from Sap1 to Sap7 were already found in drug-resistant *C. albicans* isolates. While Saps 1 to 3 act directly on tissue damage on superficial invasion, Saps 4 to 6 tend to act in deeper tissues during penetration and interact with the cellular defense [47].

*C. albicans* has developed several adaptation mechanisms in order to survive in the host organism, even in situations of pH changes and low nutrient availability [33,44,45,48]. Its high genomic plasticity allows genetic variants to better adapt to the microorganism in the environment. These mutations can affect the polymorphism, variation in chromosome copy number, recombination and total or partial loss of chromosomes under stress conditions [33]. Thus, it is possible to modulate the fungus behavior and control growth rate, morphology, and adaptation to nutrient availability. It also allows the fungus to deal with the stress induced by antifungal agents, contributing to the acquisition of resistance to drugs used in the treatment [2,33,49]. One example of this modulation is that the pH of the environment can be regulated by the fungus by excreting nitrogen, in ammonia form, promoting the growth of hyphae. Molecules such as farnesol, tyrosol and dodecanol, which act in microbial communication mechanisms, can also regulate morphogenesis by indicating cell density. When this amount is less than 10^7^ cells mL^−1^, the transition to the virulent form of hyphae occurs, being the ideal host for colonization. Contact between the fungal cell and biotic or abiotic surfaces also triggers the growth of hyphae, and on certain substrates, hyphae have the ability to invade [26,45,50]. 

Adhesion to epithelial cell surfaces is the first major virulence factor of *C. albicans*, being induced and controlled by a cascade of signals between the fungus and the host environment. Adhesion is favored by several components of the fungal cell wall, including mannose, mannoproteins and saccharins. In addition, factors such as the formation of germ tubes, the presence of mycelia and endotoxins also help with the fungus’ adhesion. On biotic surfaces, hyphae cells are capable of secreting adhesins, which allow adhesion to the epithelial cell by binding to amino acids and sugars from other surfaces, facilitating invasion [3,26,50]. 

The adhesion to abiotic surfaces is also facilitated through the formation of biofilms. Biofilm formation is an important pathogenic factor that favors cell growth and proliferation. Biofilms also offer protection from external influences, including drug treatment, because it is generally polymicrobial in nature, and the production of the extracellular matrix increases the resistance to antimicrobial therapy by preventing drug diffusion [51]. Thus, fungal biofilms are highly resistant to antifungal therapies, representing a clinical challenge, which demonstrates the importance of research aimed at its prevention and control [5]. Biofilm formation is responsible, directly or indirectly, for more than 80% of microbial infections. Besides that, the highly resistant form of biofilms is capable of facilitating the spread of the fungus in the bloodstream, leading to invasive infections in tissues and in several internal organs [1,3,44,52,53].

Two predominant mechanisms of invasion are performed by *C. albicans* cells: active penetration and induced endocytosis. In the active penetration mechanism, entry into cells is facilitated by the secretion of hydrolases capable of digesting epithelial cell surface components. These hydrolases are necessary for the disruption of host membranes and damage to the human epithelium [26,50,51,52,53,54]. Aspartic proteinase isoenzymes (Saps) are of great importance for the functionality of *C. albicans* cells, acting in adhesion processes (Saps 1-6), digestion of the epithelial cell wall, favoring invasion (Saps 2 and 9), as well as preserving the integrity of yeast cells (Saps 9 and 10). The directional growth of hyphae, called thigmotropism, helps the entry of fungal cells to host tissues by directing invasion to gaps present in epithelial cells surfaces [33,45]. In the mechanism of induced endocytosis, *C. albicans* induces the epithelial cell to promote the formation of structures similar to pseudopods, enabling the entry of the fungus by the host itself [26,50,51,52,53,54]. This induction is performed through the release of different adhesins and invasins, such as Als3 and E-cadherin. With access to epithelial cells or submucosal layers, *C. albicans* ends its pathogenicity with the induction of damage in two different ways. The fungal cell is capable of inducing apoptosis in healthy cells, through the inactivation of anti-apoptotic proteins Bcl-2 and Bcl-xL [26]. The other form of damage induction is necrosis, caused by secreted agents or associated with the hyphae of *C. albicans*. These mechanisms lead to mitochondrial edema and increase the permeability of the plasma membrane of the host cell, favoring its disruption [53,54]. 

Another important factor that needs to be highlighted here is the fact that *C. albicans* uses some metals to maintain and modulate its metabolism. Metals such as cobalt, iron, zinc, manganese, molybdenum, and copper are essential for biological processes and for structural and catalytic functions. These metals are also involved in the glycosylation and phosphorylation reactions as well as in electron transfer and oxygen transport [55,56]. Iron is one of the most relevant micronutrients during the growth and spread of *C. albicans*. Being necessary for cellular processes such as DNA replication, mitochondrial respiration and chromatin remodeling. In addition, it acts in enzymatic functions, detoxifying reactive oxygen species and in the formation of biofilms [57,58]. Besides that, iron also allows the fungus to survive in the human gastrointestinal tract as a commensal. Due to the great importance of iron for fungal metabolism, *C. albicans* has developed several metabolic mechanisms for the acquisition of the metal, being able to compete with the host for this micronutrient [58]. 

The presence of copper is also extremely important for fungal biological systems, as it offers protection against oxidative stress. In addition, copper acts as an essential cofactor in several enzymes, including energy metabolism, carbon assimilation and metabolic gene expression [55,59,60]. Zinc is essential for the functions of microbial metabolism, being the catalytic and structural center of several enzymes, and a necessary factor for fungal growth. Besides that, this metal has relevant roles in virulence factors, participating in the endothelial colonization and cells invasion of the host’s immune system by *C. albicans*. Zinc also acts in the detoxification of ROS generated by the host [61,62]. 

Although essential for fungal metabolism, metals have the ability to change their oxidation state, which can result in reactive oxygen species (from the Fenton reaction, for example) that can oxidize lipids, proteins and DNA [55]. Thus, in some situations, the accumulation of these metals can be toxic to the fungus. For this reason, the host organism can manipulate the availability of metals, decreasing or increasing them. This modulation can cause nutritional hunger or poisoning due to metal overload. *C. albicans*, on the other hand, has specific mechanisms to deal with this induced stress [60]. However, it is interesting to note that the use of these metals, associated or not with antifungals drugs, could increase their antifungal action. The association between conventional or news drugs could cause the imbalance of these metals inside the *C. albicans* cells, causing their death.

## 3. Immune Response and Conventional Treatment Therapies

The virulence mechanisms for adhesion and colonization used by *C. albicans* activate some host immunological mechanisms, necessary for successful control of the infection [51]. The presence of hyphae, essential to the pathogenic mechanisms of *C. albicans*, is the main indication of infection for the host’s immune system [26]. Epithelial cells are responsible for the initial recognition of the infection, through the detection of candidalysin. This 31-amino acid peptide presents an α-helix conformation and acts as a cytolytic toxin generated by the fungus from the Ece1 protein. Candidalysin is secreted by the hyphal portion of the fungal cell and has a cell lysis function through intercalation and permeabilization of epithelial membranes [51,52]. The detection of the toxin by the epithelial cell occurs at lower levels than the ones necessary for candidalysin inducing damage. Thus, a danger response is readily activated, leading to the initiation of innate and adaptive immunity. The recognition of candidalysin is extremely important, as it prevents benign commensal yeast cells from being unnecessarily attacked, preventing an excessive immune response that could be harmful to the host [52].

The detection of candidalysin leads to the recruitment, differentiation and activation of several immune cells. The first line of defense consists of phagocytic cells, which try to kill the fungal cell through changes in pH, potassium fluxes and activation of proteases. Besides that, the neutrophils are the main effector cells at the beginning of systemic infection and are capable of capturing and killing the fungus through extracellular traps, such as chromatin fiber networks. On the other hand, neutrophils are also able to phagocytose fungal cells, inhibiting growth and morphological transition. In addition, they can produce reactive oxygen species, and also induce the release of antimicrobial substances in the body. Macrophages are also present in the first line of defense of the immune system. They are less efficient against *C. albicans* once the phagocytosed fungal cell is able to break up the macrophage avoiding the mechanisms of death [45,63]. 

The adaptive arm of the anti-*C. albicans* involves the recruitment of dendritic cells at the target of infection. Besides killing fungal cells by phagocytosis, dendritic cells are responsible for recognizing pathogen cells and for processing an antifungal antigen, which is used by T cells to develop resistance to reinfection [63]. The release of inflammatory and chemotactic cytokines, activated by macrophages, dendritic cells and neutrophils through *C. albicans* recognition, also helps in the infection resolution [51]. As can be seen, innate immunity is extremely important to fight infections because it is the first reaction against the pathogen, preventing fungal cells from proliferating and spreading in the host organism [63]. 

Deficiencies in immune cells make the organism more susceptible to pathogens and have been associated mainly with topical skin infections. For systemic infections, the deficiency is associated with the number or action of neutrophils [5]. *C. albicans* cells also have mechanisms to neutralize the host’s immune response. Some of these strategies include secretion of hydrolytic enzymes, β-glucan protection, hyphal growth, phenotypic exchange, modulation of host T cell response and inactivation of the complement system. *C. albicans* also can induce or inhibit apoptosis, which ensures preservation, dissemination and survival of the fungal cells in the body [26,44]. 

To help the immune system to fight the infection or when the immune response alone is not enough, it is necessary to use drugs treatment. The similarity between the human epithelial cell and one of *C. albicans* cells makes it difficult to develop drugs that are not harmful to the human body [2,64]. Some classes are available for treatment and many of them have been improved over the last decades, especially in order to reduce the toxicity [3,4,64]. 

The ergosterol, a sterol similar to cholesterol, is specific to the fungal cell and is the main target for antifungal drugs. The class of azoles, which includes popular drugs such as miconazole, clotrimazole, itraconazole and fluconazole, are the most common treatments. They have fungistatic properties and can lead to the accumulation of toxic compounds in the intracellular compartment of fungal cells. Azoles act inhibiting the enzymatic activity necessary for ergosterol biosynthesis. The action occurs in the endoplasmic reticulum of the cell, causing interference in the enzyme lanosterol 14-α-desmethylase, responsible for the transformation of lanosterol into ergosterol. The low concentration of ergosterol is harmful to the structural integrity of the cell and the accumulation of 14-α-methyl-3,6-diol leads to a toxic effect for the microorganism [2,4,65,66]. The specificity of ergosterol prevents the human cell from being attacked. Although well tolerated by the human body, azole agents can present hepatotoxicity and are capable of inhibiting the action of different human enzymes [2,4].

Polyenes are another class of drugs widely used in antifungal therapy, such as amphotericin B and nystatin. These drugs break the fungal cell membrane by binding to ergosterol, causing the formation of aqueous pores along the membrane. The pores formation causes the destabilization of the cell structure and allows the leakage of intracellular content, leading to cell death [2,4,67]. Amphotericin B is one of the oldest treatments for antifungal infections, having been approved in 1957. Currently, has less toxicity than the original formulation, but it still presents some nephrotoxicity, besides the high-cost production, which makes their use less common. On the other hand, even with a slightly narrower activity spectrum than amphotericin B, nystatin is widely used, although it has some side effects and its use is not recommended for diabetic patients, due to the high level of sucrose [1,2,4,5,47].

Echinocandins are the more recently discovered class with antifungal action, being the only novel antifungal drug class to enter medical practice in decades [68]. It is composed by drugs such as anidulafungin, mycofungin and caspofungin. They are lipopeptides whose action depends on the concentration of *C. albicans* [2,65]. Echinocandins are able to damage the structural integrity of the cell wall through the non-competitive blocking of β-D-glucan synthase. Without the presence of glucan, the cell wall becomes more vulnerable to osmotic lysis [4,65,67]. Because they act on the fungal cell wall, echinocandins have a lower risk of side effects or toxicity on epithelial cells than other classes of antifungal agents [2]. As it presents a great safety profile, echinocandins were often chosen as the primary treatment of invasive candidiasis, replacing other drugs, such as fluconazole [5].

Other drugs, less common but still widely used, are flucytosine, allylamine, thiocarbamate and griseofulvin. Flucytosine, analogous to pyrimidine, is transported into fungal cells through cytosine-permease, where it acts by interfering with DNA synthesis. Flucytosine mode of action includes the inhibition of thymidylate synthase, or it can bond with RNA, interfering in translation and in protein synthesis. As a small molecule highly soluble in water, flucytosine is able to diffuse rapidly in the body when administered orally. It is a treatment rarely used as monotherapy, due to its toxic effects in high concentrations, and is generally associated with other drugs, such as amphotericin B and fluconazole [2,4,5,65]. Allylamine and thiocarbamate, on the other hand, also act by interfering with the ergosterol synthesis and affecting the integrity of the fungal cell membrane [2,65].

The increase in the occurrence of fungal infections, as well as the development of less toxic formulations of drugs, has considerably expanded the use of antifungal agents [64]. As a consequence, an increasing antifungal resistance was observed due to the adaptability shown by *Candida* species. The antifungal resistance led to an increasing in the cost and time of treatment and limits the drugs that can be used [64,69]. Microbiological resistance can occur naturally in the pathogenic fungus, without prior exposure to the antifungal agent or it can be acquired after frequent contact with the drug in question [69]. The most common mechanisms of resistance include permeability barriers (biofilms) and decreased cellular concentration of the antifungal agent. The resistance to the drug occurs when the microorganism is trying to circumvent the effects caused by the drug. The microorganism can change the molecule target or increase the transporters that remove the agent from inside the cell. Another possibility of fungus’ resistance is the adaptation to induced stress for minimizing the drug’s toxicity [2,4,67]. In the case of fluconazole, there is an up-regulation for efflux pumps capable of decreasing the intracellular concentration of the drug. Positive regulation mechanisms of the gene encoding lanosterol 14-demethylase can also occur, leading to an increase in intracellular concentration or inhibition of ergosterol formation. The replacement of the sterol by a similar molecule is another possibility for present azoles to have some effect [2,64,69,70]. Additionally, the use of fluconazole in resistant *C. albicans* can enhance the production of Saps, according to recent studies [47].

For echinocandins, resistance involves the acquisition of mutations in genes encoding catalytic glucan synthase subunits [64]. The mutations can be punctual, but they give the fungus resistance to the whole class of echinocandins. On the other hand, resistance to polyenes occurs mainly through the replacement of ergosterol by another sterol in the fungal cell membrane. These resistance mechanisms are caused by mutations in genes that encode enzymes present in ergosterol biosynthesis [2,64,65]. Resistance to flucytosine occurs with a decrease in drug uptake by cytosine permease or by changes in cytosine deaminase enzymes, that prevent the action of the antifungal agent [2]. 

The development of resistance to antimicrobial agents by microorganisms is inevitable. Factors such as the indiscriminate use of antibiotics and the current COVID-19 pandemic, as mentioned in previous topics, aggravate this situation. Thus, the need for efficient treatments and the search for new antifungal agents, which are not limited by resistance mechanisms is extremely important to eradicate the challenges associated with antifungal resistance and biofilm formation caused by *C. albicans* [4,65,71].

## 4. Histatin 5: New Antifungal Therapies

New molecules have been the target of several types of research in recent years because it is necessary to search for potential antifungal treatments that do not present resistance. These can be obtained from different sources such as natural products, synthetic agents or polymeric materials. Marine organisms, endophytic fungi, saponins, alkaloids, peptides and proteins were also investigated [65]. Peptides are molecules of great interest since they can be found naturally in the human body and some already have a function as antimicrobial agents [72]. 

Antimicrobial peptides (AMPs) display a large range of activities, being one of the first lines of defense in the human body as they are able to rapidly inhibit a broad spectrum of pathogenic microorganisms [72]. They can be isolated from prokaryotic and eukaryotic cells in the animal, plant, bacterial and fungal kingdoms. These peptides can play a fundamental role in the successful evolution of complex multicellular organisms. In general, they are characterized as amphipathic and cationic molecules with considerable variation in chain length, which can be composed of up to 50 amino acid residues and can be categorized according to their secondary structure [73].

Unlike conventional drugs, antimicrobial peptides have a low probability of acquiring resistance by microbial strains, probably due to their different mode of action [73]. The cationic properties of AMPs enable the interaction with the negative plasma membrane of the microorganism. Its affinity for the microorganism’s membrane causes an accumulation on the surface, which allows rearrangements in the membrane structure and is responsible for the translocation of the peptide for the intracellular environment. This passage across the membrane and the interactions with intracellular targets allow AMPs to bypass some resistance mechanisms [74,75]. 

Histatins are an important family of endogenous AMPs rich in histidine, that have antifungal activity against *C. albicans*, as wells as immunomodulatory, and pro-wound healing effects [76]. Therefore, it is a possible topical or systemic treatment, which can act alone or synergistically with other known drugs. Histatins are a family of small basic cationic peptides, which have a large presence of basic amino acids such as arginine, lysine and, mainly, histidine. With an isoelectric point of 6.5, histidine can modulate the cationicity of the peptide at low pH values. Its side chains are known as metal chelators, allowing the association of these peptides with metal ions [77].

Histatins peptides can be found in human saliva at concentration ranges of 50–425 μM [78]. They adopt a random conformation in aqueous solvents and α-helices structure in non-aqueous solvents. They are produced and secreted by the sublingual, parotid and submandibular glands. Secreted Histatins can undergo proteolytic degradation before reaching the mouth, and are also able to interact with other salivary molecules, becoming part of the salivary lining of hard and soft tissues [79]. These peptides were first described in the 1970s as enhancers of the glycolytic activity in some microorganisms. Around 1984, its bactericidal and fungicidal activities were described [78]. Histatins 1, 3 and 5 are the most relevant members of the family. All of them present linear structure and have 38, 32 and 24 amino acid residues, respectively, being seven of them histidines. The Hst5 primary amino acid sequence is DSHAKRHHGYKRKFHEKHHSHRGY and is characterized by a random secondary structure, presenting α-helices with only slightly amphipathic. The α-helices facilitate its entry into the pathogen’s cell cytoplasm [77]. 

Being the one with the greatest antifungal action among the Histatins, it acts against pathogenic fungi such as *C. albicans*, *Cryptococcus neoformans* and *Aspergillus fumigatus* [78]. The antimicrobial activity of Hst5 is concentrated in the region of amino acid residues located in positions 11 to 24 from the C-terminal end, called the functional domain [80]. Furthermore, the amino acids Lys13, Arg12 and Glu16 were identified through mutational analysis, as important residues for the action of the peptide [81]. Although the mechanism of action of Hst5 in *C. albicans* has not been fully elucidated, it is known that the peptide is taken up by the cell, acting intracellularly, and causes ATP efflux and production of reactive oxygen species [78]. Data presented by Moffa et al. suggest that coating oral surfaces with Hst5 in the form of a salivary film is able to reduce colonization by *C. albicans* on epithelial cell surfaces [43]. 

Although it has efficient antifungal activity when tested in vitro, Hst5 has its action reduced in the oral cavity by some interactions with metals, salts and proteins found in saliva. After its secretion, Hst5 undergoes proteolytic degradation by native enzymes present in saliva resulting in a reduction of its antifungal activity. Such proteolytic degradation represents one of the greatest challenges in the use of Hst5 as a therapeutic agent [38]. With the proteolytic cleavage, it is expected that some biological properties of the peptide also disappear. However, data presented by Helmerhorst et al. show that the initial phase of Hst5 proteolysis does not eliminate its antifungal properties. The initial degradation mixture proved to be as active as the intact peptide in antifungal assays, demonstrating that oral fluid-mediated proteolysis may be an intrinsic biological property of saliva [82]. 

Another important barrier for the use of Hst5 as a topical treatment is related to the limited activity that the peptide exhibits when present in total saliva, even in high concentrations. The processes that may help explain this factor are the binding of Hst5 with salivary salts and metals, and the dynamic turnover of salivary proteins [83]. Although the use of antifungal peptides as a treatment has a low probability of resistance development, it was found that *C. albicans* can become resistant to Hst5 after successive exposure [79]. *C. albicans* has several mechanisms to prevent death by Hst5 and is able to tolerate the presence of the peptide at low levels. *C. albicans* presents a group of Saps enzymes that is already known for causing the proteolytic cleavage of Hst5 [84]. 

Studies have shown the preference of Saps for basic or hydrophobic amino acids as cleavage sites. It was shown that Sap6, which is secreted by the fungus during the hyphae growth, reduces the Hst5 activity. The result was proven by Puri et al., through the inactivation of this enzyme by heat, who observed the non-inactivation of the activity of the peptide [35]. Bochenska et al. demonstrated that cleavage occurs first between residues K17 and H18 of Hst5 by Saps [85]. Another mechanism for *C. albicans* to avoid the action of Hst5 is binding the Msb2 protein to the peptide. Msb2 is a mucin-like sensing protein in the fungal plasma membrane, with a high molecular weight. Studies indicate that binding Msb2 to Hst5 negatively affects the activity of the peptide. In addition, the Msb2 protein also acts for stabilizing the cell wall and promoting the growth of *C. albicans* filaments [35,86].

To overcome the obstacles found in the use of Hst5 as an oral topical treatment against *C. albicans*, new mechanisms were proposed. Common ways of manipulating AMPs such as Hst5 include shortening of the peptide and amino acid substitution [72]. The introduction of unusual amino acids and modifications in the terminal regions could preserve the peptides from proteolytic degradations. In addition, reducing the size of the peptide to prevent protease attack and to decrease the cost of production is also an option. The use of efficient drug delivery systems, such as encapsulation in liposomes, for better stability and reduction of peptide toxicity, and use of tetrahedral DNA nanostructures, for their editability, biocompatibility and transportation efficiency as delivery vehicles, were also reported as a strategy to promote its use in therapy [67,87].

The 12-residue Hst5 fragment called P-113 is one of the smallest fragments with efficient activity. It has antifungal action similar to the original peptide, with high activity on strains resistant to fluconazole. Tests for modifications in amino acid residues in the structure of P-113 were performed by Rothstein et al., with the aim of improving the stability and activity of the peptide. These modifications make it a potential peptide for therapeutic use, without the obstacles found in the molecule mother [39,40]. Helmerhorst et al., as well as Lu et al., also presented some Hst5 proteolytic fragments, resulted from fungal Saps, saliva proteolysis or synthesized derivatives, that keep their antifungal activity close to or the same as the original peptide and have the advantage of not been being easily degraded [36,82,88]. 

Using amino acid modifications, Ikonomava et al., showed that substitutions of K11R-K17R residues in the Hst5 structure increased the stability of the peptide [89]. This analog can also be efficient against biofilms, once it changes one of the peptide’s main cleavage sites by Saps, and by that, reduces the biofilm viability [71]. Combinations of different peptides are also proposed to increase antifungal activity. Han et al. proposed the hybridization of Hst5 fragments with halocidin, a peptide that exerts its activity by attacking the cell membrane of *C. albicans*. All of the six hybrids generated, di-PH2, di-WP2 and HHP1, showed strong activity against different strains tested without showing cytotoxicity to epithelial cells [90]. 

It is important to consider here the association between Hst5 and some metals, such as the ones mentioned in the topic “*Candida albicans*: morphology and virulence” in this review. This association can intensify the antifungal effect of this peptide. Using the solid phase peptide synthesis (SPPS) strategy, it is possible to produce only the active fragments for this type of association. There are some studies in the literature that demonstrate this approach, such as those describe in the topic below. 

## 5. Metal Complexes and Hst5 as a Strategy to Fight *C. albicans*

Several transition metals, such as Ni, Zn, Cu, Co and Fe, as well as alkali and alkaline earth metals, such as Ca and Mg, are present in human saliva in different concentrations. Amino acids present in the sequence of Hst5, such as aspartic acid, glutamic acid, histidine, tyrosine, and serine, are known for their ability to bind to metals. Thus, 13 out 24 amino acid residues of Hst5 are potential ligands for metallic coordination, through stable complexes formed by bonds between metallic cations and coordination groups such as side chains of amino acids [83,91]. The metallic center is able to improve the peptide’s specificity, bioavailability, solubility and stability [91]. 

The properties of the Hst5-metal bond were studied for decades through in vitro investigations [92]. Such studies demonstrate the importance of coordination with metals, such as Zn (II) and Ni (II), for the secondary structure of the C-terminal region and for the α-helix conformation of Hst5. Coordination with a metallic ion can lead to differences in the interaction of the peptide with macromolecules [41,42]. The binding of metal with Hst5 can result in physiological actions related to the protection of the enamel and inhibit bacterial growth by decreasing the metal concentration. This inhibition is possible because the peptide is capable of sequestering ions necessary for microbial survival, in addition to the formation of reactive oxygen species, commonly associated with redox-active metals [92,93,94].

**Figure 1:**
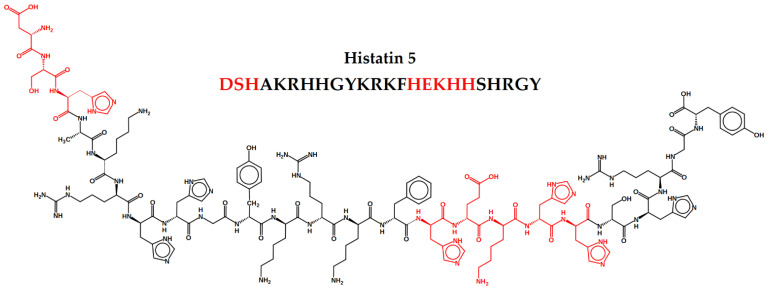
Histatin 5 structure with complexation motifs ATCUN and HEXXH highlighted, which are composed of amino acids such as histidine (H), serine (S), aspartic acid (D) and glutamic acid (E), known for metallic complexation.

Two important binding motifs were revealed by the functional and structural characterization of the N-terminal domain of Hst5: the amino-terminal DXH motif, known as ATCUN (amino-terminal copper and nickel binding unit) motif, and the HEXXH binding motif, characteristic of several metalloproteases, appears once in the Hst5 chain, and binds to Zn (II). They are both represented in the Hst5 structure in Figure 1. The dissociation constants of Hst5 with Cu (II) and Zn (II) are quite low, reaching nanomolar values, which indicates that such metals are able to bind to Hst5 motifs in saliva under physiological conditions [93,95]. The specificity loss and conformational destabilization of copper and zinc sites can be associated with a decrease in antimicrobial activity [96].

Mass spectroscopy studies show that the formation of the Cu (II)-peptide complex by the ATCUN motif is a necessary pre-requisite for the oxidative activity of Hst5 [95], being related to the increased production of reactive oxygen species after cellular uptake of Hst5, which can triplicate total intracellular levels. Copper is present in eukaryotic cells metabolism and is an important metal for cell survival [97]. The entry of Hst5 into fungal cells can lead to a competition for this metal, which can be prejudicial to the cell, even leading to cell death. Only one site for Cu (II) binding was found in Hst5, with high affinity and in a 1:1 ratio. Susceptibility tests, performed by Conklin et al., demonstrated that the binding constant was high, showing specificity between the peptide’s motif and the metal [92,93]. On the other hand, Frączyk demonstrated that serine phosphorylation may be an important mechanism of metal ion binding regulation since it weakens the stability of Cu (II) complexes [98]. Tests to assess the influence of Cu (II) ions on the antifungal activity of Hst5 showed a decrease in the EC50 value, from 5.15 μM (of Hst5 alone) to 1.36 μM, after binding to the metal [92].

Data suggest that Hst5 has three zinc-binding sites, two of them with higher affinity and one with lower affinity. It has already been discovered that Ca (II) ions interfere with peptide bonds and some metals, such as Zn (II). Thus, when the Ca (II) is present, only one of the sites with the highest affinity for zinc ions are detected, and only this one presents high selectivity [93]. Experiments carried out by Sonia Melino et al., with Hst5 in a hydrophobic environment, demonstrated the induction of peptide dimerization by zinc ions, which supports its fusogenic activity [42]. Thus, it is believed that the antimicrobial action of Hst5 can be influenced by specific molecular interactions, such as membrane aggregation by charge interaction, structural stabilization of the functional domain induced by Zn (II) and destabilization of the lipid bilayer [42]. The Hst5-Zn (II) complex also influences the antibacterial activity of the peptide, being able to induce the fusion of small negatively charged unilamellar vesicles and the hydrolysis of nucleic acids, and also increases the surface adsorption capabilities of Hst 5 at a broad pH range [95,99]. On the other hand, the binding of Zn (II) to the peptide offered little protection against proteolytic degradation [83].

Iron is one of the most abundant metallic ions in saliva, with a concentration that varies according to the diet. In the human body, it is mostly linked to other compounds, which help nourish invading pathogens. It is believed, based on the data presented by Puri et al., that the sequestration of iron by Hst5 reduces the availability of nutrients for the pathogen. However, iron-binding negatively affects the antifungal activity of Hst5 against *C. albicans*. This negative influence may result from the change in the secondary structure of the peptide, which affects the binding to the fungus cell wall [83]. Susceptibility tests have already demonstrated the complete loss of Hst5 antifungal activity in the presence of Fe (III) [92]. 

Circular dichroism studies show that Hst5 can bind up to 10 iron equivalents, and the increase in iron concentration in the structure is inversely proportional to the antimicrobial activity of the peptide. The results of Puri’s studies also demonstrated changes in the iron absorption genes of *C. albicans* treated with Hst5, showing that the binding of the peptide to iron may also contribute to a mechanism of death interfering with cellular iron metabolism. It is also possible that Hst5 can bind to intracellular iron, redistributing the cell’s reserves and leading to malfunction of the cellular perception of the metal level. The mitochondrial dysfunction in the Hst5 death mechanism can be explained by the location of iron redistributed around the mitochondria, an organelle that is extremely sensitive to metal [83].

Due to the affinity with Ni (II) in the ATCUN motif, it is expected that the metal is interacting with Hst5 in the human mouth [100]. The binding to the metal can have an impact on the peptide’s conformation increasing the stability of the α-helix, which can induce a significant difference in the peptide’s interaction with other macromolecules. The binding with Ni (II) can facilitate the Hst5 and DNA binding by locating all positive side chains on one side of the molecule [41]. However, there is still no certainty about the influence of Ni (II) ions on the antimicrobial activity of Hst5. 

On the other hand, calcium has shown to be a major inhibitor of the antifungal activity of Hst5 against *C. albicans*, at physiological concentrations. It may be the ion responsible for the decrease Hst5 detection in saliva. The inhibition of the binding between Hst5 and *C. albicans* appears to be one of the main effects demonstrated by extracellular Ca (II) [101]. Studies performed by Dong et al. showed that not only was there a reduction of up to 90% in fungal cell death in the presence of Ca (II) but also the efflux of ATP was interrupted. Similar studies were performed for salivary anions Cl^-^, CO^3-^ and for Mg (II). For the Cl^-^, CO^3-^, there was no reduction in the binding between the peptide and the fungal cell, but there was a decrease in ATP efflux [101]. 

In the presence of magnesium, there was an inhibition of up to 40% of death by Hst5, and a reduction in ATP efflux of approximately 40%. The inhibitory effects resulting from the presence of metallic cations were more pronounced than the effect for anions. The interference of calcium is much higher than the interference of magnesium, which still presents a minimal inhibitory effect within the physiological concentration ranges. The additional effect of dissociation between Hst5 and Ca (II) suggests that instead of binding to the peptide, the ion interrupts its binding to *C. albicans* cells [101]. The information about metallic complexation effects is summarized in Table 1.

The association between metal and Hst5 has a wide diversity and variation, as described here. Several metals demonstrated improvements in the original peptide, being marked as potential antifungal agents with a great interest for study and development. However, it is not yet a largely explored research area. Although it is possible to find studies about Hst5 associated with metals, not all of them explore the antifungal activity against fungal pathogens such as *C. albicans*. There are several other metals associations that can be made with Hst5, as it has many amino acids in its chain that are able to bind to metals, another possibility is the binding to other motifs present in the Hst5 amino acid chain not yet widely studied. It is also important to consider other possibilities, such as the SPPS for new peptides fragments and analogs based on the Hst5 original chain. These are just a few possibilities to be explored that can bring interesting results to help overcome the problem of antimicrobial resistance.

## 6. Conclusions

As described here, the growing increase in antimicrobial resistance, possibly intensified by the COVID-19 pandemic, has caused worldwide concern about its consequences. *C. albicans*, being the main cause of fungal diseases, must be monitored carefully. Although it is mainly associated with mild topical cases of the disease, *C. albicans* is already resistant to important drugs such as fluconazole, nystatin and amphotericin B. The resistance to these drugs favors the evolution of simple cases for more severe infections, which are associated with high morbidity and mortality rates. The increase in the use of drugs that no longer have the expected efficacy can aggravate the resistance problem. The similarity between fungal and human cells impairs the development of new drugs, that must be more specific for fungal cells and not cause toxicity to human organisms. 

The use of peptides as antimicrobial agents is promising, and Hst5 has already shown good action against *C. albicans* cells. However, the peptide has disadvantages in its stability in physiological environments, such as the oral cavity, and some mechanisms of resistance to its action can already be observed in fungal cells. The search for improvements in Hst5 action already includes several methodologies, such as the exchange of amino acid residues and the use of fragments of the original peptide. The association of Hst5 with metallic ions is an alternative not yet widely explored, but already very promising for certain metals. 

There is a greater number of studies describing the binding sites between peptide and metal, as well as the modulation of their activity after association. However, few studies investigate the antifungal capacity of Hst5 after binding to a wide variety of metals. Many of these metals are present in the oral cavity, such as Ni, Zn, Cu, Co and Fe, and can naturally bind to Hst5. For this reason, one important strategy is a wide investigation of Hst5 antifungal activity after the binding to metals. 

In summary, we present here some possibilities for using Hst5, some of them include a change in the amino acid chain, development of hybrid peptides and synthesis of smaller fragments. All these possibilities can promote the increase in the antifungal potential of Hst5. However, the focus here is the association between Hst5 and metals, producing metallopeptides. This is a field with several possibilities to be explored, that certainly could contribute to the development of new antifungal therapies that can overcome the resistance barriers presented by *C. albicans*.

## Figures and Tables

**Table 1:** Summary of the main findings of the effects on Histatin 5 by the metallic complexation.

Metal	Coordination Site in Hst5	Complexation Effects	Reference
Copper	ATCUN	Improves peptide stability Enhances antifungal activity Raises ROS liberation Enhances the fungus nutritional hunger	[92] [93] [95]
Zinc	HEXXH	Improves peptide’s functional domain stability Improves the stability of peptide’s α-helical conformation Destabilizes fungus lipidic bilayer Promotes fungus acid nucleic hydrolysis Little protection on peptide proteolytic degradation	[42] [83] [95]
Nickel	ATCUN	Improves the stability of peptide’s α-helical conformation Facilitates the peptides bond with fungus DNA	[41] [100]
Iron	uncertain	Minimizes drastically antifungal activity Enhances the fungus nutritional hunger Affects the peptides interaction with the fungal cell wall Interferes on iron cellular metabolism, leading fungal mitochondria to death	[83] [92]
Calcium	uncertain	Inhibits antifungal activity Suppresses ATP efflux Interrupts the bond between peptide and fungus cell	[101]
Magnesium	uncertain	Minimizes antifungal activity Decreases ATP efflux	[101]
